# Protocol for a pilot trial to assess the feasibility of the Move More @ Work intervention to encourage employees to take the opportunity to move (be physically active) after every 30 min of sitting

**DOI:** 10.1186/s40814-021-00903-2

**Published:** 2021-09-07

**Authors:** Elaine A. Hargreaves, Jillian J. Haszard, Sally Shaw, Meredith C. Peddie

**Affiliations:** 1 School of Physical Education, Sport and Exercise Sciences, University of Otago, PO Box 56, Dunedin, New Zealand; 2 Department of Human Nutrition, University of Otago, PO Box 56, Dunedin, New Zealand

**Keywords:** Sedentary behaviour, Prolonged occupational sitting, Regular activity breaks, Behaviour change, Workplace health promotion, Accelerometry, Interrupted time series design

## Abstract

**Background:**

Prolonged sitting increases the risk of cardio-metabolic disease. Office-based employees are particularly susceptible to high rates of this sedentary behaviour during work hours. Laboratory studies indicate that regularly interrupting periods of prolonged sitting with short bouts (2 min) of physical activity can improve markers of cardio-metabolic health. This method of interrupting sitting time is yet to be tested in an occupational setting and may provide an alternative to providing sit-to-stand desks. Drawing on the Behaviour Change Wheel and evidence on the barriers and motivators to performing regular activity breaks, the Move More @ Work intervention was developed. The objectives of this pilot study are to examine the feasibility, and preliminary outcomes, of this intervention designed to encourage participants to perform 1–2 min of activity after every 30 min of continuous sitting throughout the work day. The study will inform if progress to a full effectiveness trial is warranted.

**Methods:**

An interrupted time series design consisting of a 4-week baseline (control period), a 12-week intervention, and a 12-week follow-up will be utilised. At least 57 university employees who self-report spending > 5 h per day sitting at work on at least 3 days per week will be recruited to participate. The intervention consists of (1) a structured consultation with a Move More @ Work coach, containing a number of behaviour change techniques to create an individualised plan of how to incorporate the activity breaks into the working day, and (2) strategies to create a supportive workplace culture for performing the activity breaks. Feasibility will be assessed by recruitment and retention rates, and acceptability of the intervention. Pilot outcomes are the number of regular activity breaks taken during the workday, cardio-metabolic risk score and self-reported health, and work-related productivity outcomes.

**Discussion:**

If the Move More @ Work intervention is shown to be feasible, acceptable, and shows evidence of effectiveness, this will provide justification for the progression to a full scale evaluation of the intervention. In the longer-term, this intervention may provide an alternative means of improving health outcomes through interrupting sedentary time than that offered by current sedentary behaviour interventions.

**Trial registration:**

Australian New Zealand Clinical Trials Registry, ACTRN12620000354987. Registered on 12 March 2020

**Supplementary Information:**

The online version contains supplementary material available at 10.1186/s40814-021-00903-2.

## Background

Sedentary behaviour — seated or reclined activities involving low energy expenditure [1] — predominate the waking hours of most adults. Total sedentary behaviour is accumulated across the day as a consequence of a number of life events, e.g., watching TV, car travel, and work-related tasks. Sustained bouts of sitting have been associated with increased incidence of diabetes, cardiovascular disease, some cancers, and overall mortality [2–4]. Those in office-based employment are particularly susceptible to high rates of this sedentary behaviour during work hours [5]. Observational studies have shown that the pattern in which total sedentary time is accumulated also influences these negative outcomes. Individuals who accumulate their sedentary time in long uninterrupted bouts (periods > 60 min) have a poorer cardio-metabolic risk factor profile (higher waist circumference, body mass index [BMI], fasting glucose and triglyceride concentrations) than those whose total sedentary time is the same but is accumulated in short bouts interspersed with light or moderate intensity activity [6, 7]. These associations appear to be consistent across age, sex, and ethnic subgroups [6], and occur even in those who engage in large amounts of leisure time moderate-to-vigorous physical activity [6, 7]. Additionally, at least in older women, those who have the longest bouts of uninterrupted sedentary time are 22% (95% CI, 1 to 46%) more likely to develop cardiovascular disease compared to those with shorter bouts [8]. Tightly controlled laboratory or office-based studies have shown that regularly interrupting periods of prolonged sitting (every 20–30 min) with a short bout of activity (2 min) results in improvements to postprandial glucose and lipid metabolism in healthy adults [9, 10] and those with obesity and type 2 diabetes [11]. Importantly, when prolonged sitting is interrupted with only short bouts of standing, the results on postprandial metabolism are smaller in magnitude and less consistent [9, 10].

The majority of field-based intervention studies that aim to reduce occupational sitting have provided individuals with standing, or sit-to-stand workstations. A systematic review and meta-analysis of the use of activity-permissive workstations [12] reported decreases in sedentary behaviour of up to 1.6 h over an 8 h work day, and some modest improvements in markers of cardio-metabolic health. An intervention where participants take the opportunity to perform short (1–2 min) bouts of activity (e.g., walking, simple body weight resistance exercises) to interrupt periods of prolonged sitting daily over a period of weeks and months has the potential to successfully attenuate the risks associated with prolonged sitting. This novel intervention addresses the call for much needed, well-designed, evidence-based research to reduce sitting time [13] and has the potential to dramatically extend knowledge in the field of sedentary behaviour by providing an alternative to the provision of standing desks as a means to interrupt sedentary time. Yet to substantiate these claims, the intervention must first be subject to a feasibility study to test the research methods, implementation, and acceptability to participants and provide an indication of effectiveness [14]. The feasibility study will effectively answer the question, ‘will the intervention increase the number of short bouts of activity people perform at work?’ Previous research has typically used the term ‘regular activity break’ to describe these short bouts of activity taken at regular intervals during the day. From here on, and throughout our intervention, we will refer to these breaks as ‘opportunities to move’, rather than activity breaks. Through our preliminary research on workplace culture conducted in preparation for the intervention, senior management expressed concern that the term ‘activity break’ may be misconstrued by employees as a contracted break offered as part of an employment contract. Consequently, we want to avoid using the term ‘break’.

The feasibility of making sustained behaviour changes from prolonged sitting to taking regular opportunities to move in an occupational setting has not been studied. Like most behaviour change interventions, a key factor is understanding if individuals will adhere to the intervention and how best to motivate them to perform this activity. A recent systematic review of behaviour change strategies used in interventions designed to reduce general sitting time concluded that interventions performed to date have given insufficient attention to motivation [13]. This review found self-regulatory skills (self-monitoring of behaviour, action and coping planning, feedback), education, and environmental restructuring were important strategies to support behaviour change [13]. To specifically explore factors that would support and hinder the ability to take an opportunity to move and provide essential knowledge for the development of this Move More @ Work intervention, we conducted focus group research with office-based employees [15]. Our work found that behavioural prompts, rewards, the ease of incorporating opportunities to move into the work day and having external support were important strategies to facilitate behaviour change. In conjunction, we found a number of social and individual barriers that need to be overcome including, perception of lowered work productivity, workplace social norms and culture, and type of work task [15]. These have also been shown to be barriers to reducing sitting time [16, 17]. To increase the individual’s perceived ability to take an opportunity to move and reduce their sedentary behaviour, interventions need to employ these behaviour change techniques and employ strategies to overcome the unique barriers people face [13].

In designing the Move More @ Work intervention, we drew on the Behaviour Change Wheel [18] and its associated COM-B model (Capability, Opportunity, Motivation and Behaviour [18];) and theoretical domains framework (TDF [19];) to provide an integrated synthesis of the key behaviour change constructs and specific behaviour change techniques that would provide the mechanisms of action for individuals to incorporate more movement into their work day. This comprehensive framework is instrumental to inform intervention design and evaluation [18] and has been used in other sedentary behaviour interventions [17]. The COM-B model identifies that to encourage people to take opportunities to move requires improved capability, opportunity, and motivation. The TDF represents key concepts from behaviour change theory that can be used to structure the important components required when implementing an intervention (e.g., knowledge, goals, environmental context, and resources).

## Objective

The objective of this pilot study is to assess the feasibility, and potential effectiveness, of the Move More @ Work intervention designed to encourage staff to take 1–2-min opportunities to move after every 30 min of prolonged sitting over the work day. An opportunity to move is defined as greater than 1 min of light to moderate activity involving repeated movement of some kind (e.g., brisk walking, body weight resistance exercises, or moving through a series of stretches). The study will inform if progress to a full effectiveness trial is warranted. The specific aims of this study are the following:

1. Evaluate the recruitment rate of participants into the study, and retention of those participants through the intervention and post-intervention period.
2. Qualitatively examine the acceptability of the Move More @ Work intervention to participants, workplace management, and where possible, employees not involved in the intervention but working in close proximity to those that were.
3. Evaluate the extent to which Move More @ Work changes the number of opportunities to move taken over the work day and the extent of participant adherence to the intervention.
4. Examine the potential effects of Move More @ Work on Cardio-Metabolic Risk score (an estimated risk based on waist circumference, blood pressure, fasting glucose, triglycerides and High Density Lipoprotein [HDL] cholesterol), musculoskeletal health, psychological well-being, and perceptions of work productivity.
5. Use the findings to refine the intervention and inform the development of a subsequent effectiveness trial of the Move More @ Work intervention.
6. Estimate the sample size required in a subsequent effectiveness trial evaluating the intervention.

## Methods

### Study design and setting

An interrupted time series design will be utilised in this study [20, 21]. With this design, there will be no control group but instead there will be two pre-intervention assessments, a post-intervention assessment and a follow-up assessment 12 weeks post-intervention. This quasi-experimental design can be used instead of a randomised controlled design to estimate an intervention effect in situations where randomisation is not feasible. In this case, the interrupted time series design is used to avoid the high risk of contamination between a control group and an intervention group, where these groups could be in close working proximity. The study will be conducted at the University of Otago, Dunedin, New Zealand. In this large workplace open-plan offices are commonplace, movement between offices is common, and it is possible that while taking opportunities to move, intervention group participants could mix with control group participants. Data will be collected between August 2020 and February 2021.

**Fig. 1:**
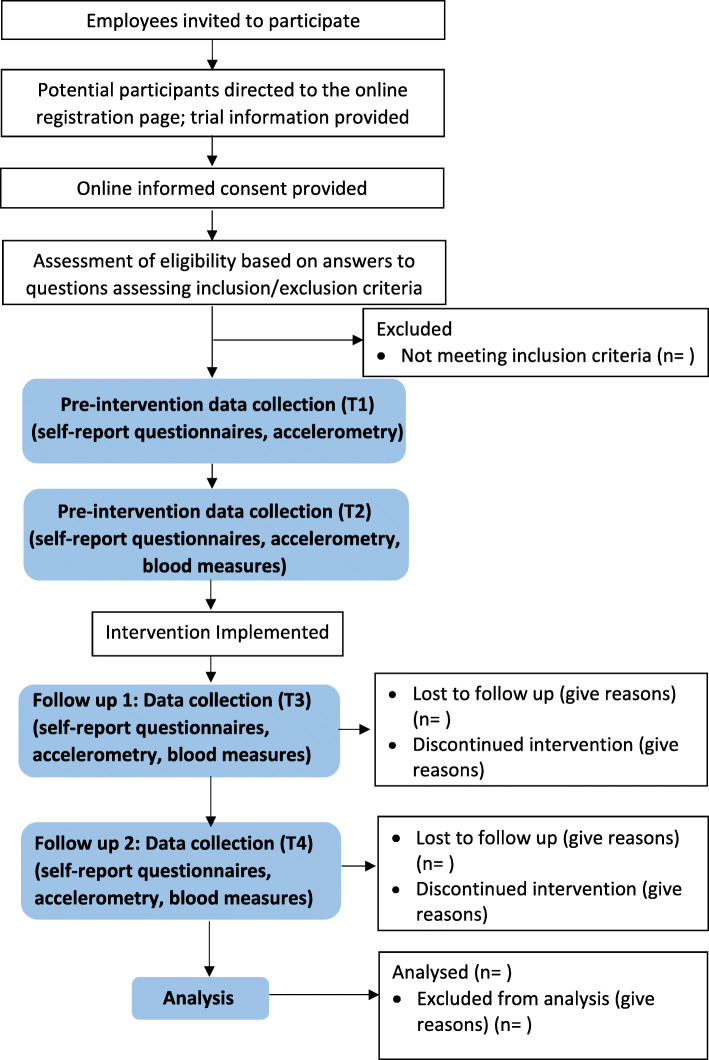
Flow diagram of the Move More @ Work feasibility and pilot trial

There will be four time points for data collection (not all variables will be collected at all time points — see “Outcomes” section below for details): Baseline (T1) and 3 weeks later at time 2 (T2) which corresponds to the pre-intervention period, at completion of the 12 week intervention (T3), and then after the 12 week post-intervention follow-up period (T4). Figure 1 shows the study flow diagram. This study has been approved by the University of Otago Human Ethics Committee (ref H20/028). This protocol has been prepared in accordance with the Standard Protocol Items: Recommendations for Interventional trials (SPIRIT) 2013 statement [22], the SPIRIT schedule of enrolment, interventions, and assessments is presented in Table 1. The SPIRIT Checklist can be found in Additional File 1. This study is registered with the Australian New Zealand Clinical Trials Registry: ACTRN12620000354987 (Additional File 2). Protocol version 2 dated June 2020.

**Table 1:**
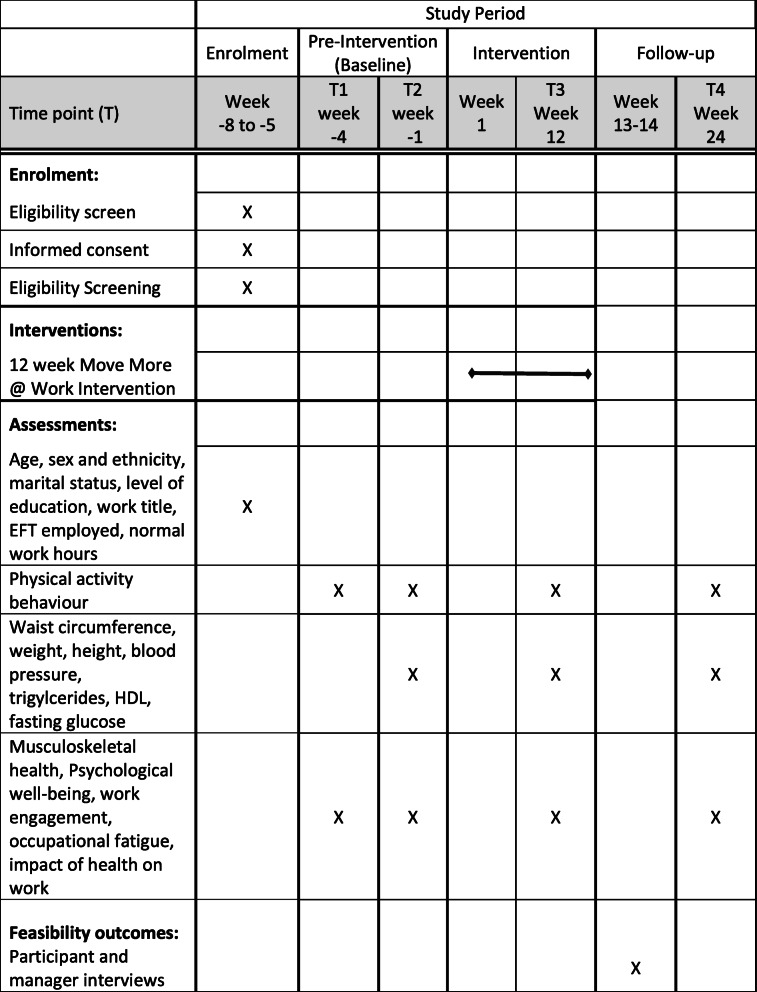
The schedule of enrolment, interventions, and assessments for the Move More @ Work study using an interrupted time series design

### Outcomes

#### Feasibility

Drawing from recommendations [23], feasibility will be assessed through recruitment and retention rates assessed at pre-intervention through to T4. Acceptability will be assessed through participant feedback on satisfaction with the conduct of the intervention, and usefulness of the specific intervention components collected at T3.

#### Pilot study primary outcome

The primary outcome is workday physical activity behaviour, specifically, the change in the mean number of opportunities to move taken during a workday from the pre-intervention period (T1 and T2) to the end of the intervention (T3) and post-intervention (T4) periods. An estimated change will be calculated, along with an estimate of the within-person variability, which can then be used in sample size calculations for future studies.

#### Pilot study secondary outcomes

Secondary outcomes are health- and work-related outcomes. Specifically, cardio-metabolic risk score will be measured at pre-intervention T2 and assessed by changes from T2 to the end of the intervention (T3) and post-intervention (T4) periods. Results of any change in health outcomes between T2 and T3 will be used to inform the sample size for a future effectiveness trial which will likely have health variables as the primary outcome.

Finally, musculoskeletal health, psychological well-being, occupational fatigue, the impact of health on work, and physical activity patterns outside of work will be assessed by changes from the pre-intervention period (T1 and 2) to the end of the intervention (T3) and post-intervention (T4) periods.

### Participant inclusion and exclusion criteria

Participants will be drawn from academic, professional and administrative employees at the University of Otago. To be eligible for inclusion, participants must (1) be older than 18 years; (2) self-report spending > 5 h per day, on at least 3 days of the week in a seated position at work (objectively measured sedentary time of participants who self-report being sedentary at work, has been reported to be 5.5 h [5]); and (3) not use a sit-to-stand desk. Part-time employees are eligible providing they meet the criteria above. Participants will be excluded from participating if (1) they have any physical or physiological impediments to participating in physical activity because this study requires participants to undertake short bouts of physical activity (all participants will be screened for contra-indications using the Physical Activity Readiness Questionnaire [PAR-Q] [24] prior to starting the study), (2) they have a planned absence from work for > 2 weeks during the intervention period, or (3) they plan to relocate to another workplace other than the University of Otago, Dunedin campus, during the 6 month study period (intervention and follow-up). Participants will also be excluded from participation if they are pregnant, as pregnancy has the potential to affect waist circumference, and other components of the cardio-metabolic risk score (a secondary outcome measure).

### Recruitment

Information about the study and copies of the recruitment flyers advertising the study will be emailed directly to contact points in all University Departments, and posted on campus related social media sites. Paper copies will be posted around the university campus and information on the study included in the Otago Bulletin Board (the University’s online newsletter). Individuals will either register their interest to participate by emailing the study investigators or will register directly on the link provided in the recruitment email.

### Sample size

From a feasibility study perspective, a formal sample size calculation is not required. However, for the pilot study component, one stated aim is to investigate changes in the number of opportunities to move taken over the work day (by comparing data from the pre-intervention to post-intervention periods) and the extent of participant adherence to the intervention. Consequently, using the standard deviation in the number of opportunities to move observed in previous research [5], our sample size calculation indicate that 47 participants from approximately 10 different offices will provide 90% power to detect a difference of three opportunities to move between pre-intervention and completion of the intervention or post-intervention periods to an alpha level of 0.05. This assumes that the within-person correlation in activity breaks is 0.5 and the standard deviation is 3. To account for office clusters a design effect of 4.2 was used (using an average cluster size of *n* = 5 and a strong ICC of 0.8). Allowing for a drop-out of rate of ~ 20%, 57 participants will be recruited. Having this stated sample size will also allow us to investigate the feasibility of achieving our target recruitment goal.

### Study procedures

#### Participant registration

Participants will confirm their interest in being involved in the study by registering online. A link will be sent to the participant to complete a questionnaire through REDCap, an online data capture system [25]. Here, participants will read the information sheet, provide consent, and then complete screening questions to assess eligibility. Participants can also request a paper copy to complete if preferred. Participants who do not meet the eligibility criteria will be informed of this and thanked for their interest. Those eligible will go on to provide demographic information (age, sex and ethnicity, marital status, level of education, work title, EFT employed, normal work hours), questionnaire-based outcome measures (e.g., psychological well-being, occupational fatigue, musculoskeletal health), and will be scheduled for an assessment session to complete the pre-intervention biophysical outcome measures.

#### Pre-intervention period

In this interrupted time series design, we will assess participants’ usual pattern of physical activity behaviour over a 4-week period prior to the intervention being introduced. We have identified this as the pre-intervention period and will assess accelerometer-measured physical activity after participants have registered for the study (T1) and 3 weeks later (T2). This essentially controls for any effects that may arise as a result of being in a study and any potential trends in physical activity across time. At the conclusion of this pre-intervention period, the intervention will be introduced.

### Move More @ Work intervention

The aim of this 12-week intervention is to encourage participants to perform 1–2 min of activity after every 30 min of continuous sitting throughout their workday. The intervention contains an individual behaviour change component delivered by a Move More @ Work Coach and a workplace culture component. The Behaviour Change Wheel [18] and the associated COM-B model and TDF were used in the design of the intervention along with research evidence of behaviour change strategies important to support reduction in sedentary behaviour [13] and our work investigating facilitators and barriers to performing regular activity breaks [15]. The specific intervention components and their mapping to the COM-B model and TDF are shown in Table 2.

**Table 2:** Links between the COM-B model, TDF domains, intervention functions, and behaviour change techniques used in the Move More @ Work Intervention

COM-B model component and definition of the barrier/motivator influencing change		TDF domains	Intervention functions	Behaviour change techniques	Description of intervention strategy
Capability	*Psychological* A lack of knowledge of why prolonged sitting is harmful to health and benefits of incorporating regular movement.	*Knowledge* Increase knowledge of health risks to prolonged sitting (even when regularly physically active) and benefits of reduced sitting time. *Beliefs about consequences* Create expectancy that positive outcomes will occur with reduced sitting.	Education, persuasion	• Information on health consequences • Credible source	• Information on health consequences discussed by the Move More @ Work coach (MMWC) and in study booklet provided to participants.
	*Psychological* A lack of awareness of how sedentary the individual currently is.	*Knowledge* Increase awareness of how sedentary the individual currently is.	Education, persuasion	• Feedback on behaviour • Discrepancy between current behaviour and goal	• Participant booklet displays individualised pre-intervention activity patterns at work and expected behaviour during intervention. • Consultation with MMWC discusses discrepancy between current and expected behaviour and elicits participant’s feelings about discrepancy to encourage motivation to change.
	*Psychological/physical* A lack of understanding of what physical activities meet the criteria and how to perform them.	*Knowledge* Increase knowledge of what physical activities can be performed during an opportunity to move. *Skills* Develop skills necessary to perform required activities safely.	Education, training, persuasion	• Instruction on how to perform the behaviour and demonstration of the behaviour	• Examples of activities, and demonstration videos, that meet the requirements of an opportunity to move provided in study booklet. • MMWC demonstrates the behaviours during consultation.
	*Psychological* Lack of confidence in being able to perform regular opportunities to move.	*Beliefs about capabilities* Increase confidence in being able to change sedentary behaviour. *Goals* Create action plans. *Memory, attention, and decision processes* Enable decisions to move rather than be sedentary. *Behavioural regulation* Identify success through self-monitoring of behaviour.	Enablement, training, environmental restructuring	• Action planning • Problem solving • Prompt/cues • Self-monitoring of behaviour	• MMWC explains how to set action and barrier-coping plans. • Weekly email encourages participants to complete action and barrier-coping plans and to send a copy back to research team. • A reminder prompt set up on participant’s computer. • Participants provided with a daily checklist to tick off every time an opportunity to move is taken. This is returned to research team at the end of the week.
Opportunity	*Social* Perception that workplace culture created by managers is unsupportive of taking opportunities to move.	*Social influences* Create a workplace culture where managers are supportive of taking breaks.	Environmental restructuring, modelling	• Information about others’ approval • Restructuring the social environment • Social support (unspecified) • Identification of self as role model	• Support and encouragement for taking opportunities to move communicated at daily/weekly team meetings with manager. • Senior-managers endorse the study and encourage staff to take part through staff newsletters and email communications. • Managers encouraged to role model taking opportunities to move.
	*Social* Perception that colleagues would not be supportive of taking opportunities to move.	*Social influences* Create a social norm around taking opportunities to move.	Environmental restructuring	• Information about others’ approval • Restructuring the social environment	• Support and encouragement for taking opportunities to move communicated at daily/weekly team meetings with manager. • Staff display information on their desk stating, ‘I am taking an opportunity to move, I will be back in 2 min’.
	*Physical* Requirement for personalised support to enact change.	*Environmental context and resources* Provision of personalised support to develop required skills and abilities to take an opportunity to move.	Environmental restructuring, enablement	• Prompts/cues • Social support (Practical)	• A reminder prompt set up on participant’s computer. • Individualised plan created in meeting with MMWC.
Motivation	*Reflective* Overcoming perceptions that taking opportunities to move will decrease work productivity and not provide short-term benefits.	*Beliefs about consequences* Believing that taking breaks will not decrease productivity and will provide benefits. *Reinforcement* Recognising consequents of action will provide reinforcement for behaviour.	Education incentivisation, enablement	• Information on health consequences • Self-monitoring of outcomes of behaviour • Monitoring of emotional consequences	• Information presented by MMWC and in study booklet. • Participants asked to reflect at the end of each week about how they feel.
	*Reflective* Lack of motivation to perform the behaviour.	*Intentions* Create a conscious decision to change behaviour. *Goals* Create action plans to support intentions. *Behavioural regulation* Identify success through self-monitoring of behaviour.	Enablement	• Action planning • Problem solving • Self-monitoring of behaviour	• Weekly email encourages participants to complete action and barrier-coping plans and send a copy back to research team. • Participants provided with a daily checklist to tick off every time an opportunity to move is taken. This is returned to research team at the end of the week.
	*Automatic* Support to create a new habit and break old habits.	*Reinforcement* Create a new habit and routine.	Enablement	• Habit formation • Prompts/cues	• An opportunity to move is repeatedly taken following the reminder prompt set up on computer.

#### Move more @ work coach consultation

All participants will have an individual consultation with a Move More @ Work coach lasting around 30 min. The coach will undergo training on how to deliver the intervention components and to ensure that all elements are delivered as intended. Participants will be provided with a hard copy of a study booklet outlining the points discussed by the coach. Specifically, during this consultation, the coach will do as follows:

1. Briefly, discuss current evidence around sitting as a health hazard, and the health benefits associated with performing regular short bouts of movement throughout the day.
2. Discuss and demonstrate where necessary, examples of physical activities that meet the intensity and duration required of an opportunity to move (for example, walking up and down stairs or along a corridor; repeatedly standing up from the chair and sitting back down; body weight resistance exercises such as squats, calf raises). Links to online videos demonstrating these specific movements will be provided in the study booklet.
3. Discuss the participant’s profile of the time they spent sitting and being active at work collected from the pre-intervention accelerometer data. The coach will highlight the discrepancies between current sedentary time and the expected behaviour if participants take an opportunity to move every 30 min.
4. Develop an individualised action and barrier-coping plan [26]. The action plan involves participants specifying exactly what activities they will perform (and would easily fit into their work context), when they will do them (e.g., may have different activities at different times of the day), and where (e.g., in the office, on the stairs). The barrier-coping plan involves participants identifying potential barriers that they may face when implementing this plan and putting strategies in place to overcome those barriers. A laminated card will be provided for the participant to record their action and barrier-coping plan each week. They will be encouraged to place this card within eyesight of their workstation to assist with self-monitoring.
5. Provide participants with a laminated chart to record each opportunity to move that was performed each work day. At the end of each day, a section on the chart will prompt participants to self-reflect on how they are feeling.
6. Discuss which external electronic prompt the participant would like to use as a reminder to take their opportunities to move (e.g., an outlook calendar reminder — available on a PC or Mac, or the computer announcing the time every 30 min — Mac only). The coach will then help the participant set these prompts up on their computer.

At the beginning of each week of the intervention period, participants will receive an email reminding them to set weekly action and barrier-coping plans. Participants will be asked to respond to the weekly email by sending back a copy of their action and barrier-coping plan to the research team (to indicate they have completed their plan). They will also be asked to email back a copy of the chart which detailed the number of opportunities to move performed on each day of the previous week.

#### Workplace culture

Workplace culture and management-level support is essential to the adoption of any individual-based intervention [27]. To facilitate the creation of a supportive workplace culture, senior and mid-level university managers were interviewed to ascertain their views on the study being conducted. Importantly, they were asked for input into the strategies that could be employed to communicate their support and encouragement to employees who chose to participate. Managers were highly supportive of the intervention being put in place with the caveat that guidelines/boundaries were put in place to sustain work productivity and limit distraction to others. Consequently, communications will be created for dissemination by senior management to employees that state that participation in the study will be supported and the direct managers will be encouraged to reiterate their support during weekly team meetings. Communications will also be sent to Heads of Department to explain that the study is running and to support the study messages in case any of their staff have opted to take part.

### Measures

#### Feasibility: recruitment, retention, and acceptability

Successful recruitment will be defined as recruiting at least 57 participants over 1 month. Successful retention will be deemed as retaining 80% of participants who complete pre-intervention measurements (T1 and 2) through to the measurements taken at post-intervention follow-up (T4).

At the end of the intervention period (T3), semi-structured interviews will be held with participants, managers, and where possible, those who did not participate in the intervention but were working in close proximity to those that were. The purpose of the interviews is threefold. First is to ascertain why participants volunteered and their experiences of participating in the Move More @ Work intervention. Here, we will examine perceptions of how the intervention was delivered by the coach, the usefulness of the behaviour change components included in the intervention and the extent to which a supportive culture was created with respect to taking opportunities to move. We will also examine the acceptability of the data collection processes (wearing the accelerometers, blood collection, etc.). Second is to examine the impact of the intervention of those who did not actively participate. Third is to identify managers’ perspectives of employee participation in the intervention and perceptions of its strengths and weaknesses. These data will inform subsequent changes to the recruitment into, and delivery of, the intervention in any future trial. Participants who drop out of the intervention will be followed-up where possible to ascertain reasons for dropping out.

### Pilot study outcomes

All measures (except blood-related measures) will be taken pre-intervention T1 and T2, end of the 12-week intervention (T3), and after a 12 week follow-up period (T4).

#### Change in physical activity behaviour

The number of opportunities to move that have been taken and 24 h patterns of physical activity will be identified using output from two accelerometers worn for 24 h a day for seven days at each assessment period. The ActiGraph Gt3x+ accelerometer is worn on an elasticated strap around the waist so that the accelerometer sits over the right hip (which provides accurate information about intensity of activity). The ActiGraph will be initialised to record activity at a sampling rate of 30 hz without the low-frequency extension. The ActivPAL3 is attached to the midpoint of the front of the right thigh with an adhesive dressing (which provides accurate information about posture). Prior to attachment, the ActivPAL will be inserted into nitrile two finger cots secured using a piece of adhesive to ensure it is water proof.

During each 7-day wear period, participants will complete a wear time diary, in which they will record times when either accelerometer was removed for more than 10 min (to identify non-wear time, e.g., for swimming or playing contact sport), times of day spent at work, and the time they attempted to fall asleep at night and got up in the morning.

ActiGraph data will be saved in 15-s epochs and data from both the ActiGraph and ActivPAL will be uploaded into Stata for processing. Non-wear time and hours spent at work will be identified based on times reported in the wear time diary. Days will only be considered valid if: wear time during waking hours is ≥ 10 h, and total wear time is ≥ 20 h. A participant’s data will only be included in analysis if it contains more than three workdays of valid data. Using the ActiGraph data, time spent sedentary will be classified as < 150 cpm using the *y*-axis [28]. Time spent in light and moderate-to-vigorous physical activity will be identified using a threshold of 150 to 1951 cpm and at least 1952, respectively, using the *y*-axis thresholds [29]. Time spent asleep will be identified using the Sadeh algorithm [30] constrained by the bedtimes reported in the wear time diary. The activPAL data will be used to identify time spend sitting, standing, and stepping that will be presented in parallel to the ActiGraph data. An opportunity to move will be defined as an increase in counts per minute to over 1000 cpm (ActiGraph data), when the participant is not seated (activPAL data) that is sustained for more than a minute (adjustments will be made if any participants have issues with mobility). Our previous work [31] indicates that the 1000 cpm threshold is associated with the intensity of activity shown in laboratory studies to produce clinically meaningful reductions in postprandial glucose and insulin concentrations. To avoid clusters of activity being identified as multiple breaks, at least 15 min of sedentary time must have been accumulated between opportunities to move. Total and work time spent in moderate-to-vigorous physical activity, light activity, sedentary behaviour, and sleep will be identified.

Self-reported adherence to the intervention will also be collected. Participants will record the number of opportunities to move taken each work day and will send the weekly record to the research team by email at the start of the following week.

#### Cardio-metabolic risk score

Cardio-metabolic risk assessment will be undertaken at pre-intervention T2. This assessment comprises measures of waist circumference, weight, height, blood pressure, triglycerides, HDL, and fasting glucose. Waist circumference will be measured to the nearest 0.1 cm with a steel measuring tape at the midpoint between the lowest rib and the iliac crest. Weight will be measured to the nearest 0.1 kg with the participant wearing light clothing and no shoes using calibrated body weight scales. Height will be measured to the nearest 0.1 cm, without shoes, with the individual looking straight ahead (Frankfort plane), using a calibrated stadiometer. All anthropometric measurements will be taken in duplicate, with a third measurement taken if the first two differ by more than 0.5 units. Blood pressure will be measured via a digital blood pressure monitor (OMRON HEM-907; Omron Healthcare, Japan) using the right arm and appropriately sized cuff. Participants will rest in a seated position for 15 min prior to having three measurements taken at 1-min intervals. Fasting blood samples will be collected at the Department of Human Nutrition Research Clinic in the morning by a trained phlebotomist for the analysis of glucose and lipids (triglycerides, HDL cholesterol). Samples will be centrifuged within an hour of collection and plasma stored at – 80 °C until analysis, which will be conducted in the Department of Human Nutrition Diabetes and Lipids Laboratory. Lipid and glucose concentrations will be measured using glycerol phosphate oxidase and hexokinase enzymatic methods, respectively, on a Cobas C 311 analyser.

The overall cardio-metabolic risk score will then be calculated by first log10 transforming and normalising (mean/SD) the relevant biomarkers and then taking a weighted average of their values: 1/5 waist circumference + 1/5 triglycerides − 1/5 HDL + 1/5 fasting glucose + 1/5 mean of systolic and diastolic blood pressure. This method has been used in previous research [32].

*Musculoskeletal health* will be assessed using the Standardised Nordic Questionnaire [33]. This measures musculoskeletal problems in nine body areas (neck, shoulder, upper back, elbow, wrist, lower back, hip, knee, and ankle). Participants respond either yes or no to whether they have had experienced any trouble in each body area and, if so, how much pain was experienced on a scale of 1 to 10. A lower score signifies better musculoskeletal health. Similar to Dunstan et al. [34], the wording will be modified to refer to the last 7 days and last 3 months (instead of 12 months).

*Psychological well-being* will be assessed using the Positive and Negative Affect Schedule — short form (PANAS) [35]. This scale assesses two sub-scales: negative affect and positive affect. Participants report on a 5-point Likert-type from 1 (never) to 5 (always) the extent to which they generally feel each of the 10 items. Lower scores on the negative affect subscale and higher scores on the positive affect subscale represent better psychological well-being.

*Work engagement* will be assessed using the Utrecht Work Engagement Scale 9 [36] which comprises three sub-scales: vigour, dedication, and absorption. Participants record on a 7-point Likert scale (0 [never] to 6 [always, every day]) how frequently they have had a particular feeling described in each item. Higher scores represent greater work engagement.

*Occupational fatigue* will be assessed using the 11-item Need for Recovery Scale [37]. The scale assesses the extent to which work tasks induce a need to recuperate from work induced effort. Participants report with a yes or no to each of 11 items. The sum of the items scored as ‘no’, is multiplied by 100 and divided by 11 (the total number of scale items) resulting in an overall score between 0 and 100. A higher score reflects a greater need for recovery and increased short-term work-related fatigue.

*Impact of health on work* will be assessed using the Work Limitations Questionnaire — Short Form [38]. Participants rate their level of difficulty (or ability) to perform in eight areas of work in the past two weeks (e.g., to concentrate on work, speak with people, handle the workload, and finish on time) on a scale capturing the percentage of time they have met the specific work demand (‘all of the time (100%)’, ‘most of the time’, ‘some of the time (about 50%)’, ‘a slight bit of the time’, ‘none of the time (0%)’ and ‘does not apply to my job’). The eight items comprise four work limitation sub-scales: time management, physical demands, mental and interpersonal, and output demands. Responses are converted to percentages to create a score ranging from 0 to 100 for each subscale. Higher scores reflect greater difficulty in being able to perform their work.

### Data analysis

Descriptive statistics will be used to assess the feasibility outcomes of time taken to recruit and retention of participants across the intervention. Intervention acceptability will be assessed through analysis of participant interviews. They will be open-coded, examining for common themes in relation to acceptability and usefulness of the intervention. They will then be axially coded, investigating the relationships between the themes and the similarities and differences in individuals’ reporting and experiences of those themes [39].

The primary and secondary outcomes of the pilot study will be assessed using mixed effects regression models, where the independent variables in the model will be: time since baseline, pre-/post-intervention (dummy variable), and a time by pre-/post-intervention interaction variable. The immediate effect of the intervention will be represented by the regression coefficient of the pre-/post-intervention variable, with the regression coefficient of the interaction term representing the difference in change in the outcome over time. Because this study examines within-person changes, only participants with data at T1, T2, and T3 will be included in the primary analyses, using an intention-to-treat approach. Office clusters will be included as a random effect. Where appropriate, the Stata command ‘itsa’ (for interrupted time series analysis) will be used. Mean changes in outcomes with 95% CI will be reported. To illustrate the results of the pilot study and assist in interpretation, line graphs will be generated. No interim analysis is planned for the trial. All quantitative statistical analysis will be carried out using Stata (StataCorp, Texas).

To estimate sample sizes for a future effectiveness trial, standard deviations of the change in health outcomes (e.g., body weight, blood pressure, and lipids) between T2 and T3 will be calculated. Estimates of intraclass correlation coefficients (from office clusters) will also be used to estimate the design effect if the future trial is planned to be a cluster randomised controlled trial [40].

### Retention

Participants will receive a weekly email from the research team, which aims to promote adherence to the intervention and retention in the study. Follow-up data will be collected from all available participants irrespective of their compliance to the intervention.

### Criteria to indicate that a future effectiveness trial is feasible

Drawing on suggested criteria [23, 41] decisions on whether to proceed to an effectiveness trial is warranted will be determined on the basis of (1) meeting stated recruitment (57 participants within 1 month) and retention (retaining 80% of participants from pre-intervention to post-intervention follow-up) targets; (2) participants expressing that the conduct of the intervention was acceptable, that the components contained with the intervention were useful, and that any implementation barriers experienced by participants can be overcome; (3) that at least 50% of the sample recorded an average of at least 8 (half of the possible 16 in an eight hour day) opportunities to move at the end of the intervention; and (4) within and between estimates of variation in health indicators of interest can be observed, to inform the appropriate sample size calculation for an effectiveness trial.

### Data management

Due to the low risk nature of the trial, a data monitoring committee was not deemed necessary. All self-reported data will be directly input via online surveys to the web-based data capture system REDCap hosted at the University of Otago. All objective data collected at face-to-face sessions (e.g., weight, height, blood pressure) will be collected on hardcopy and then input to REDCap. Data entry will be double checked. The REDCap database that contains the study data represents each participant by an ID number and will be password protected. Accelerometer data will be saved in files identified by an ID number and will be saved on a password protected server. Only the research team will have access to the dataset. The de-identified information collected as part of this research will be retained for at least 10 years in secure storage.

### Adverse events

The intervention is not expected to result in any serious harm or adverse event. Participants will be asked to report any physical activity-related serious adverse events to the principal investigator. In the unlikely event that an injury is sustained, then participants will be covered by New Zealand’s Accident Compensation Corporation scheme.

## Discussion

Prolonged bouts of sitting have been associated with increased incidence of diabetes, cardiovascular disease, some cancers, and overall mortality [2–4]. Those in office-based employment are particularly susceptible to both high rates of sitting and accumulating that sitting time in long uninterrupted bouts during work hours [5]. Laboratory studies have shown the benefits to health when periods of prolonged sitting are interrupted every 20–30 min with a short (2 min) bout of activity [10, 11]. The Move More @ Work study will provide the next level of evidence for the benefits of this protocol by examining its feasibility and potential effects in a real world setting.

Our Move More @ Work intervention is underpinned by key behaviour change constructs and techniques known to motivate behaviour change [18, 19] and address the key barriers and facilitators [13, 15–17] that influence the ability to take opportunities to move and reduce sedentary behaviour. By investigating work productivity and manager/work colleague opinions of the participant’s involvement in the intervention, we provide evidence of its ‘real world’ applicability. If the study meets the stated progression criteria this will provide evidence that an effectiveness trial is warranted [42] and that the intervention has the potential to successfully attenuate the health risks associated with prolonged sitting in an at-risk population.

## Supplementary Information

**Additional File 1:.** SPIRIT 2013 Checklist: Recommended items to address in a clinical trial protocol and related documents.

**Additional File 2:.** Move More @ Work Trial Registration Data.

## Data Availability

The datasets used and analysed during the current study are available from the corresponding author on reasonable request.
