# Supplementary material for: Protocol for a pilot trial to assess the feasibility of the Move More @ Work intervention to encourage employees to take the opportunity to move (be physically active) after every 30 min of sitting
Source: Pilot Feasibility Stud. 2021 Sep 7;7:172. doi: 10.1186/s40814-021-00903-2 (PMC8422613; doi:10.1186/s40814-021-00903-2)
Supplement: Supplementary file 2 — Additional File 2:. Move More @ Work Trial Registration Data. [file 40814_2021_903_MOESM2_ESM.pdf]

Move More @ Work Registration Data	
**Data Category**	
**Primary Registry and Trial Identifying Number**	Australia New Zealand Clinical Trials Registry<br>ACTRN12620000354987
**Date of Registration in the Primary Registry**	4th March 2020
**Secondary Identifying Numbers**	
**Source of Monetary or Material Support**	University of Otago Research Grant
**Primary Sponsor**	University of Otago
**Secondary Sponsor**	
**Contact for Public Queries**	Meredith Peddie<br>move-more-at-work@otago.ac.nz
**Contact for Scientific Queries**	Principle Investigator: Elaine Hargreaves<br>(03) 479 8941<br>Elaine.hargreaves@otago.ac.nz<br><br>Principle Investigator: Meredith Peddie<br>(03) 479 8358<br>meredith.peddie@otago.ac.nz
**Public Title**	Move more at work: A feasibility and pilot study
**Scientific Title**	Move more at work: A feasibility and pilot study of university staff taking opportunities to move
**Countries of Recruitment**	New Zealand
**Health Condition(s) or Problem(s) Studied**	Sedentary Behaviour
**Intervention(s)**	The aim of this intervention is for participants to perform 1-2 min of activity after every 30 min of continuous sitting throughout their work day. Once both pre-intervention assessments have taken place, all participants will have an individual consultation with a Move More @ Work Coach (a postgraduate student trained in physical activity related behaviour change techniques) lasting around 30 min. This consultation will mark the start of the intervention period. During this consultation, the coach will:<br>1. Briefly, discuss current evidence around sitting as a health hazard, and the health benefits associated with performing regular short bouts of movement throughout the day.<br>2. Discuss, and demonstrate where necessary, examples of activities that meet the intensity and duration required of an opportunity to move. These activities will also be detailed in the participant booklet that accompanies

	the study. Links will also be provided to videos showing the specific movements being undertaken.<br>3. Discuss their individual profile of the time they spent sitting and being active at work (collected from the accelerometers) during the pre-intervention assessments. The coach will highlight the discrepancies between current sedentary time and the expected behaviour if participants take an opportunity to move every 30 min.<br>4. Develop an individualised action and contingency plan (Bélanger-Gravel A, Godin G, Amireault S. A meta-analytic review of the effect of implementation intentions on physical activity. Health Psychology Review 2013;71:23-54). The action plan involves participants specifying exactly what activities they will perform (and would easily fit into their work context), when they will do them (e.g., may have different activities at different times of the day), and where (e.g. in the office, on the stairs). The barrier contingency plan involves participants identifying potential barriers that they may face when implementing this plan and putting strategies in place to overcome those barriers (Bélanger-Gravel A, Godin G, Amireault S. A meta-analytic review of the effect of implementation intentions on physical activity. Health Psychology Review 2013;71:23-54).<br>5. Provide participants with a laminated chart to record that the opportunity to move specified in their action plan was performed. This will also provide the prompt for participants to self-reflect on how they are feeling both physically and mentally at the end of each day. Participants will be asked to report the number of opportunities to move recorded on this chart back to the research team on a weekly basis by responding to the weekly study email.<br>6. Discuss which external electronic prompt the participant would like to use as a reminder to take their opportunities to move (an outlook calendar reminder -available on a PC or Mac, or the computer announcing the time every 30 min –Mac only). The coach will then help the participant set these prompts up on their computer.<br><br>A personalized booklet outlining these discussion points will be provided to each participant in hard copy (designed specifically for this study). Participants will also receive a laminated card on which to record their weekly action and contingency plan. Participants will be encouraged to place their action and contingency card within eyesight of their workstation to assist with self-monitoring.<br><br>At the beginning of each week of the intervention period, participants will receive an email reminding them to set weekly action and barrier plans. Participants will be asked to email a photo of this plan at the beginning of the week (to indicate they have completed their plan) to the research team.
**Key Inclusion and Exclusion Criteria**	Inclusion Criteria: Employees of the University of Otago who are based at the Dunedin campus, who are older than 18 years and who self-report sending >5 h per day, on at least three days of the week in a seated position at work.<br>Exclusion Criteria: Participants will be excluded from participating if they have any physical or physiological impediments to participating in physical activity (as identified by the Physical Activity Readiness Questionnaire). Participants will also be excluded if they have a planned absence from work

	for >2 weeks during the study, or if they plan to relocate to another workplace during the study. Participants will be excluded from participation if they are pregnant.
**Study Type**	Interventional<br>Allocation: Non randomized interrupted time series design<br>Assignment: Control followed by intervention<br>Purpose: Treatment
**Date of First Enrollment**	06/07/2020
**Sample Size**	Target Sample Size: 57
**Recruitment Status**	Yet to begin
**Primary Outcome**	The change in number of opportunities to move that have been taken during the work day, measured via accelerometry at baseline (weeks -4 and -1) and the end of the intervention (week 12) and the end of the follow up period (week 24)
**Key Secondary Outcomes**	Total physical activity, sedentary time and sleep measured via accelerometry at baseline (weeks -4 and -1) and the end of the intervention (week 12) and the end of the follow up period (week 24).<br>Cardio-metabolic risk score (made up of measurements of waist circumference; measured using a anthropomorphic measuring tape, fasting glucose; measured in plasma using the hexokinase enzymatic method, fasting triglyceride; measured in plasma using the glycerol phosphate oxidase enzymatic method and, systolic and diastolic blood pressure measured via automated sphygmomanometer), measured at baseline (week -1) and the end of the intervention (week 12) and the end of the follow up period (week 24).<br>Musculoskeletal health will be assess using the standardized Nordic Questionnaire, Psychological Well-Being will be assessed using the Positive and Negative Affect Schedule – short form (PANAS), Work Engagement will be assessed using the Utrecht Work Engagement Scale 9, Occupational fatigue will be assessed using the 11-item Need for Recovery Scale and impact of health on work will be assessed using the Work Limitations Questionnaire – Short Form, measured at baseline (weeks -4 and -1) and the end of the intervention (week 12) and the end of the follow up period (week 24).
**Ethics Review**	Status: Approved<br>Date: 17/02/2020
**Completion Date**	Anticipated date of last data collection: 19 February 2021
**Summary Results**	Analyses have not been conducted yet.
**IPD sharing statement**	De-identified individual participant data underlying published results will be available on a case by case based at the discretion of the primary investigators, who can be contacted by email using the address above.
